# Domoic Acid Improves the Competitive Ability of *Pseudo-nitzschia delicatissima* against the Diatom *Skeletonema marinoi*

**DOI:** 10.3390/md11072398

**Published:** 2013-07-11

**Authors:** Emily K. Prince, Friederike Irmer, Georg Pohnert

**Affiliations:** 1 Department of Mathematics, Sciences, and Technology, Paine College, 1235 15th Street, Augusta, GA 30901, USA; 2 Institute for Inorganic and Analytical Chemistry, Friedrich Schiller University, Lessingstrasse 8, Jena 07743, Germany; E-Mails: friederike.irmer@gmx.de (F.I.); georg.pohnert@uni-jena.de (G.P.)

**Keywords:** iron, *Pseudo-nitzschia*, harmful algal bloom, competition, domoic acid, allelopathy

## Abstract

Because domoic acid, a neurotoxic secondary metabolite produced by marine diatoms in the genus *Pseudo-nitzschia*, is hypothesized to be part of a high affinity iron uptake system, we investigated whether domoic acid could improve the competitive ability of *Pseudo-nitzschia delicatissima*, and whether the availability of iron changed the outcome of competition experiments. We found that domoic acid had a slight negative effect on growth of the diatom *Skeletonema marinoi* when it was grown in monocultures. However, when *S. marinoi* was cultured with *P. delicatissima* the presence of domoic acid resulted in a reduction of *S. marinoi* cells by up to 38% and an increase in *P. delicatissima* cell numbers by up to 17% under iron replete conditions. Similar effects were not observed in low iron treatments. Domoic acid was not taken up by *P. delicatissima* cells. Overall, our results indicate that domoic acid can improve the competitive ability of *Pseudo-nitzschia* spp. and that iron is likely to be involved. This study provides an unusual example of indirect inhibition of competitor growth mediated by a secondary metabolite.

## 1. Introduction

Iron has received increased attention for its role in governing phytoplankton growth and abundance in offshore high nitrate-low chlorophyll areas (HNLC) [1,2,3] as well as in some coastal upwelling regions [4]. Large scale iron fertilization experiments have shown conclusively that iron limits phytoplankton growth in 30% to 40% of the world’s oceans [5]. These fertilization experiments, and resulting increase in phytoplankton biomass, have been the center of a debate over whether induced phytoplankton blooms might enable the long-term remove of CO_2_ from the atmosphere, a process that has the potential to ameliorate global climate change [6,7,8]. Regardless of the potential to influence climate processes, iron fertilization experiments have underscored the importance of iron as a limiting nutrient in planktonic communities and have highlighted the link between iron utilization and ecological processes.

Phytoplankton have evolved a variety of mechanisms for accessing iron in the ocean. For example, some pennate diatoms, including *Pseudo-nitzschia* spp. and *Fragilariopsis* spp., contain ferritin, an iron concentrating protein, which enables them to store iron if this resource is available only intermittently [9]. Additionally, because more than 99% of the dissolved iron in the ocean is chelated with organic ligands [10,11], phytoplankton have evolved mechanisms to successfully access bound iron. Recently, iron bound to saccharides has been reported to be available to eukaryotic algae [12]. Iron may also be bound by siderophores, compounds released to facilitate iron uptake by complexation, or by porphyrin, which is largely released upon cell degradation. Eukaryotic phytoplankton outcompete bacteria in accessing this second class of chelated iron, but are unable to easily take up iron bound to siderophores [13]. However, eukaryotic algae also form mutualistic relationships with bacteria, providing other nutrients in exchange for siderophore-bound iron [14].

The pennate diatoms in the genus *Pseudo-nitzschia* may have an additional mechanism to tolerate iron limitation. *Pseudo-nitzschia* spp. produces the neurotoxic compound domoic acid, which is responsible for amnesiac shellfish poisoning (ASP) in humans, and has devastating effects on seabirds and marine mammals [15]. Domoic acid has been shown to bind iron [16], leading to speculation that it may be part of an iron uptake mechanism [17,18]. However, results from studies of domoic acid production under iron limitation are mixed. Although some studies have shown that domoic acid production decreases dramatically in low iron conditions [19], more recent studies have shown that, while the amount of intracellular domoic acid does decrease, the amount of dissolved domoic acid increases in low iron cultures. These results indicate that iron limitation may increase the release of domoic acid into the media [17]. When media of *Pseudo-nitzschia* spp. were supplemented with additional domoic acid, the uptake of iron was increased by a factor of three, further supporting the idea that domoic acid plays a role in iron uptake [17]. However, because domoic acid binds to iron with lower affinity than do bacterial siderophores [16], it is unlikely that it could compete with these compounds at concentrations commonly found in nature [18]. Instead, evidence supports the hypothesis that domoic acid chelates copper, which may then be used in a high affinity iron uptake system [18].

Most studies of the role of domoic acid in iron uptake have occurred in monocultures of *Pseudo-nitzschia* spp., which provide valuable information but cannot account for the complexity of natural systems. Although domoic acid added to monocultures of other phytoplankton species had no effect on their growth [20], the question of whether domoic acid can change the outcome of competition between *Pseudo-nitzschia* spp. and competitors remains unanswered. However, recent shipboard experiments do suggest that domoic acid may have an effect on phytoplankton competition. Trick *et al.* [21] added iron or domoic acid to two distinct natural oceanic phytoplankton communities. In both cases iron addition resulted in an increase of chlorophyll a, however, the resulting increase could be explained by an increase in diatom abundance in only one of the communities. In contrast, the addition of domoic acid increased the chlorophyll a concentration and as well as the concentration of diatoms, likely by increasing the relative availability of ambient iron. In all cases, *Pseudo-nitzschia* spp. dominated the diatom community [21]. It remains unclear, however, whether domoic acid addition improved the competitive ability of *Pseudo-nitzschia* spp. relative to other diatoms, or if increased concentrations of domoic acid increased iron availability for all diatom species.

In the present study we investigate how domoic acid influences the outcome of competition between *Pseudo-nitzschia delicatissima* and the cosmopolitan marine diatom *Skeletonema marinoi* under low iron and iron replete conditions. We hypothesized that, while domoic acid would have no effect on *S. marinoi* grown alone, in co-cultures domoic acid would increase the relative abundance of *P. delicatissima*. We were able to test the effects of domoic acid on co-cultures using an isolated *P. delicatissima* strain previously reported to produce domoic acid, but which did not contain this metabolite under our culture conditions, allowing the direct manipulation of domoic acid concentration. Our findings indicate that domoic acid does improve the competitiveness of *P. delicatissima* in iron replete, but not low iron culture conditions. These results suggest that domoic acid may play an important role in structuring phytoplankton communities.

## 2. Results and Discussion

### 2.1. Effects of Domoic Acid on Algal Monocultures

This study benefitted from the use of a strain of *P. delicatissima* reported to produce domoic acid by the Provasoli-Guillard National Center for Marine Algae and Microbiota (NCMA) but which did not produce domoic acid over the course of our experiment, ensuring that the concentration of domoic acid in culture could be directly manipulated. Because this strain was previously reported to produce domoic acid, it is likely that it retained the ability to utilize domoic acid. It is unclear why *P. delicatissima* produced no domoic acid in our study, however low irradiance levels used in this experiment have been reported to reduce domoic acid production [22].

**Figure 1:**
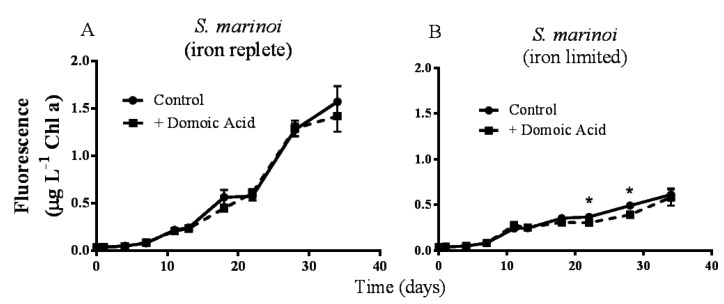
Effects of 482 nM domoic acid on monocultures of *S. marinoi* under iron replete (**A**) and low iron (**B**) conditions (Experiment 1). Graphs show the growth of *S. marinoi* control cultures (solid line, filled circles) and cultures to which domoic acid was added (dashed line, filled squares). Error bars indicate standard error. Asterisks (*) indicate a significant difference between treatments (*p* < 0.05) (*n* = 5 for all).

Experiment 1 determined the effect of domoic acid on algal monocultures. *Pseudo-nitzschia delicatissima* chlorophyll a fluorescence was not significantly different between control cultures and cultures to which domoic acid had been added, either under iron replete (*p* = 0.272) or under low iron culture conditions (*p* = 0.603; data not shown). Similarly, the fluorescence of *Skeletonema marinoi* cultures was unaffected by domoic acid under iron replete (*p* = 0.141) conditions (Figure 1A). However, under low iron conditions a slight, but significant effect of domoic acid on *S. marinoi* cultures was detected (*p* < 0.001). Subsequent post-hoc tests determined that the fluorescence of low iron *S. marinoi* cultures was significantly less in the presence of domoic acid on day 22 (17.7 ± 4.74% reduction; *p* = 0.010) and on day 28 (20.1 ± 4.23% reduction; *p* < 0.001) (Figure 1B). This effect was no longer observable by day 34 (*p* = 0.224).

Analysis of domoic acid from the *P. delicatissima* and *S. marinoi* cultures in experiment 1 indicated that the concentration in the samples did not decrease over the course of the experiment (*p* = 0.471). Additionally, the concentration of domoic acid measured in the cultures was never significantly different between any of the treatments (*P. delicatissima* or *S. marinoi*, with or without iron) (*p* = 0.083) (data not shown).

In contrast to previous reports that demonstrate no allelopathic effect of domoic acid [20], our results suggest that a domoic acid at high concentrations may very slightly inhibit the growth of *S. marinoi*. However, the previous study considered phytoplankton grown under nutrient replete conditions. The low iron treatment may have increased the susceptibility of *S. marinoi* to other stressors, in this case, domoic acid. Previous studies have shown that nutrient limitation increases the susceptibility of phytoplankton to radiation [23,24] and to unidentified allelopathic compounds produced by other phytoplankton [25]. From this experiment, it is not clear whether domoic acid directly inhibits the growth of *S. marinoi* or if domoic acid binds iron, and reduces iron availability to the already iron limited *S. marinoi* cells. If domoic acid reduces iron availability for *S. marinoi*, it may explain why the effects of domoic acid disappear by day 34. The exudation of many *S. marinoi* metabolites increases in the declining culture phase [26]; these metabolites may include iron containing compounds and lysis of cells might release iron as well. If this iron is available to *S. marinoi* the growth of iron limited cultures exposed to domoic acid may recover by the end of the experiment.

It should be also noted that the concentrations of domoic acid used in experiment 1 were quite high (approximately 482 nmol L^−1^), and much higher than the concentrations used in subsequent experiments (from 6.34 to 63.4 nmol L^−1^). In order to determine whether domoic acid ever has an effect on *S. marionoi* monocultures, we chose to test its effects with concentrations higher than those reported in *Pseudo-nitzschia* spp. blooms. Although concentrations of >100 nmol L^−1^ have been reported in blooms [16] these values are not commonly reached in the field. In contrast, domoic acid can reach concentrations more than an order of magnitude higher than those used here [19,27] in cultures of *Pseudo-nitzschia* spp. However, even in situations where the concentration of domoic acid in water samples is low, local maxima might occur. Because domoic acid exuded by *Pseudo-nitzschia* spp. will exist in patches of high concentrations surrounding the producing cells [28,29], it is possible that *S. marinoi* may encounter domoic acid at concentrations used in this study. However, suppression of growth is modest, and whether such effects really provide an advantage under natural conditions remains to be established.

### 2.2. Effects of Domoic Acid on Algal Co-Cultures

**Figure 2:**
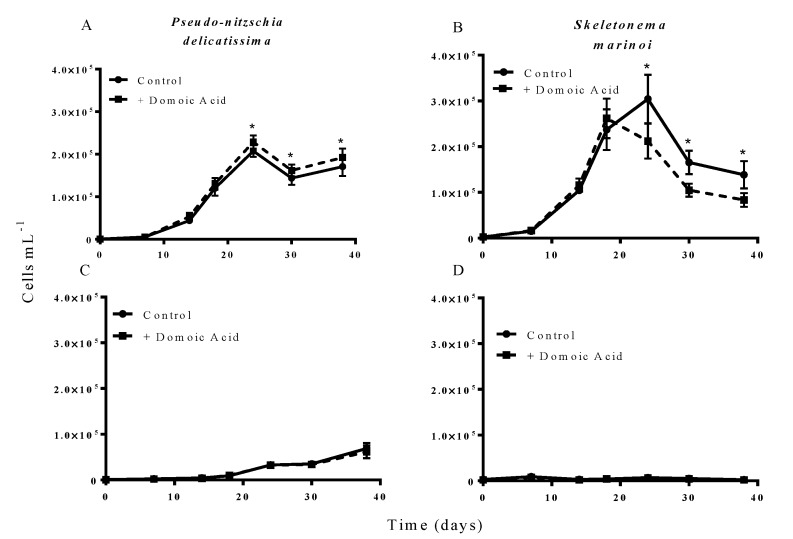
Effect of 64 nM (high) domoic acid on co-cultures of *S. marinoi* and *P. delicatissima* (Experiment 2). Graphs show cell numbers of *P. delicatissima* (**A**) and *S. marinoi* (**B**) under iron replete conditions as well as cell numbers of *P. delicatissima* (**C**) and *S. marinoi* under low iron conditions (**D**). Cell numbers in control cultures (solid line, filled circles) and cultures to which domoic acid was added (dashed line, filled squares) are depicted in each graph. Error bars indicate standard error. Asterisks (*) indicate a significant difference between treatments (*p* < 0.05) (*n* = 7 for all).

The results from experiment 2, testing the effect of domoic acid in situations where the two phytoplankton species directly interacted, indicated that domoic acid has the potential to increase the abundance of *P. delicatissima* and to decrease the abundance of *S. marinoi* in co-cultures. When co-cultures were grown under iron replete conditions, the addition of “high” (64 nM) domoic acid concentrations marginally but significantly increased growth of *P. delicatissima* (*p* = 0.016) (Figure 2A). From day 22 until the end of the experiment on day 38 the number of *P. delicatissima* cells in co-cultures with domoic acid was between 10.6% and 17.4% higher than in cultures with no additional domoic acid (*p* = 0.004–0.012). There was no significant effect of the addition of “high” concentrations of domoic acid on the growth of *S. marinoi* in co-cultures under iron replete conditions (*p* = 0.087). However, we did detect a significant interaction of time and treatment (*p* = 0.003), suggesting that *S. marinoi* growth curves were different between co-cultures with and without domoic acid. Closer inspection revealed that between day 22 and day 38, the concentration of *S. marinoi* cells was between 23.9% and 37.6% lower in the treatment that received domoic acid when compared to the treatment that did not receive domoic acid (*p* = 0.024 to <0.001) (Figure 2B). In contrast, there was no significant effect of domoic acid addition on the growth of either *P. delicatissima* (*p* = 0.465) (Figure 2C) or *S. marinoi* (*p* = 0.089) (Figure 2D) in low iron co-cultures. Similarly, there was no significant interaction for *S. marinoi* or *P. delicatissima* cells between the low iron treatments with and without domoic acid and time (*p* = 0.972, and *p* = 0.855, respectively).

**Figure 3:**
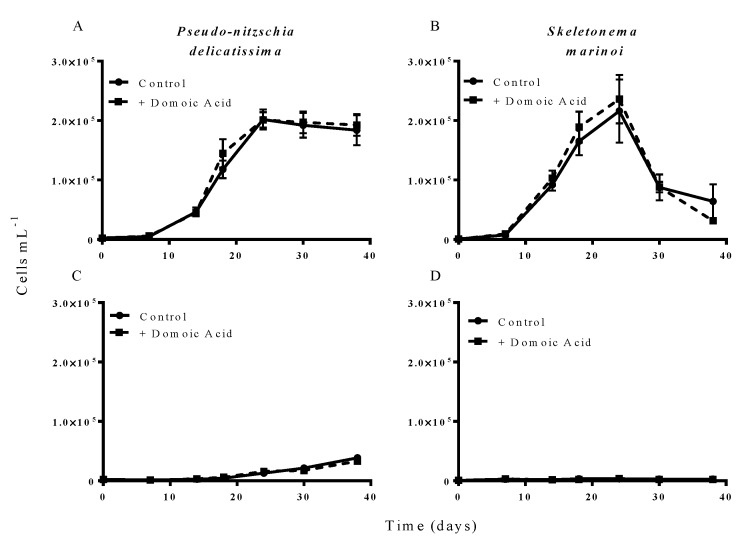
Effect of 6.4 nM (low) domoic acid on co-cultures of *S. marinoi* and *P. delicatissima* (Experiment 2). Graphs show cell numbers of *P. delicatissima* (**A**) and *S. marinoi* (**B**) under iron replete conditions as well as cell numbers of *P. delicatissima* (**C**) and *S. marinoi* under low iron conditions (**D**). Cell numbers in control cultures (solid line, filled circles) and cultures to which domoic acid was added (dashed line, filled squares) are depicted in each graph. Error bars indicate standard error. No significant differences were detected between treatments (*n* = 7 for all).

Additions of “low” (6.4 nM) concentrations of domoic acid did not influence the growth of either *P. delicatissima* or *S. marinoi* under either low iron or iron replete conditions. *P. delicatissima* growth under iron replete conditions was not significantly different with or without added low domoic acid concentrations (*p* = 0.259) and there was no significant interaction between treatment and time (*p* = 0.559) (Figure 3A). Likewise, *P. delicatissima* growth under low iron conditions was not significantly different between cultures with added domoic acid and without (*p* = 0.626) and there was no significant interaction between of treatment and time (*p* = 0.221) (Figure 3C). Similarly, the growth of *S. marinoi* was not different between treatments with and without domoic acid under iron replete (*p* = 0.808) or low iron conditions (*p* = 0.158). There was no significant interaction between treatment and time for *S. marinoi* cells in either low iron (*p* = 0.224) or iron replete cultures (*p* = 0.325) (Figure 3B,D).

When domoic acid was added to cultures at a concentration of 64 nmol L^−1^, the growth of *P. delicatissima* was slightly stimulated while the growth of the competitor, *S. marinoi*, was more dramatically inhibited, with the final ratio of *P. delicatissima* to *S. marinoi* to be 2.3 ± 0.4 in the treatment containing iron and domoic acid, and only 1.2 ± 0.3 in the comparable treatment without domoic acid (Figure 2A,B). The fact that similar effects were not observed under low iron conditions (Figure 2C,D) might be due to the fact that iron concentrations were too low to support *S. marinoi* growth even in the absence of domoic acid, a possibility supported by the weak growth of *S. marinoi* monocultures in iron limited media (Figure 1B). This hypothesis could also explain why no differences in *S. marinoi* cell densities were detected until day 22 of experiment 2, after *P. delicatissima* reached stationary phase, since iron concentration was unlikely to be limiting, even with the addition of domoic acid, before this point (Figure 2B). The results from experiment 2 are in contrast to the results from experiment 1, which found domoic acid had an effect on *S. marinoi* only in low iron conditions (Figure 1A). We hypothesize that the difference is caused by the presence of *P. delicatissima* in the co-cultures used in experiment 2, which used iron in the media, reducing concentrations in the iron replete media to a level at which domoic acid had an effect. In addition, *P. delicatissima* may have reduced the iron concentrations in low iron media below the level required to support *S. marinoi* growth, so that the addition of domoic acid did not have any effect.

While our results do establish that domoic acid can improve the competitive ability of *P. delicatissima*, it is less clear whether this effect is related to iron availability. We propose two alternate hypotheses. First, domoic acid prevents *S. marinoi* from iron acquisition, either by directly binding the metal ions, as we suggested for *S. marinoi* monocultures (Figure 1B), making them inaccessible for *S. marinoi* or by facilitating iron uptake by *P. delicatissima*. Alternately, domoic acid could improve the competitive ability of *P. delicatissima* through a mechanism that does not involve iron and the differences between the effects of domoic acid on low iron and iron replete cultures could be caused by differences in the physiological state of diatom cells. However, this second hypothesis is less likely given previous reports of the interactions between domoic acid and iron in blooms and cultures of *Pseudo-nitzschia* spp. [16,18,21].

The fact that domoic acid stimulated the growth of *P. delicatissima* only modestly under iron replete conditions, but not at all under low iron conditions (Figure 2A,C) is in contrast to previous results, which have shown a marked increase in *Pseudo-nitzschia* cell numbers in response to domoic acid [18,21]. However, these studies have considered natural assemblages of the phytoplankton community. Sources of iron in these samples are likely to have been diverse, consisting primarily of iron bound to organic ligands and may be accessible for *Pseudo-nitzschia* through domoic acid [10,11]. In contrast, iron in our experiment was added to replete cultures as FeCl3 with an equivalent concentration of EDTA. Although additional sources of iron in both replete and limited cultures likely included impurities present in other trace metals and contained in the seawater used to make media, it is unlikely that the medium contained significant amounts of iron bound to the array of bacterial siderophores, porphyrin-complexes, and other organic ligands present in natural phytoplankton communities [13]. It is likely that the iron in this experiment was already available to *P. delicatissima*, limiting the advantage to be gained from the presence of domoic acid. Concentrations of domoic acid of more than 100 nmol L^−1^ have been detected during *Pseudo-nitzschia* blooms in Monterey Bay, California [16]. Therefore, the domoic acid concentrations used in this experiment, 64 and 6.4 nmol L^−1^ are ecologically relevant. We could reliable quantify approximately 0.6 nmol L^−1^ domoic acid, but were able to detect significantly lower concentrations. Although it is not possible to determine whether the strain of *P. delicatissima* used in this experiment produced very low levels of domoic acid, it is clear that *P. delicatissima* produced domoic acid at concentrations much lower than the low domoic acid treatment used in this experiment. Moreover, we detected no domoic acid at any time point in any treatment unless it was directly added to the cultures.

**Figure 4:**
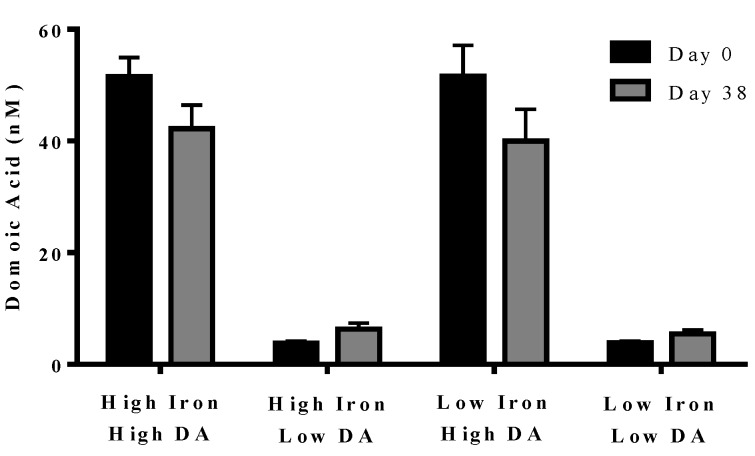
Concentration of domoic acid in “high” and “low” domoic acid treatment co-cultures on day 0 (black bars) and day 38 (grey bars) (Experiment 2). No significant differences were found between iron replete and low iron treatments, nor were difference detected over time. Error bars indicate standard error (*n* = 7 for all).

In experiment 2, domoic acid was measured at the beginning (day 0) and end (day 38) of the experiment. No domoic acid was detected in any of the cultures, either with or without iron, unless it was added separately (data not shown). Domoic acid concentration in the treatment did not change over the course of the experiment (*p* = 0.210). The domoic acid concentration was higher in high domoic acid than in low domoic acid treatments (*p* < 0.001 in all cases). Domoic acid concentration was not different between low iron and iron replete cultures for high domoic acid treatments (*p* = 0.624–0.748) or low domoic acid treatments (*p* = 0.850–0.941) (Figure 4). These results indicate a negligible turnover and degradation of domoic acid in phytoplankton cultures.

### 2.3. Domoic Acid Is Not Taken up by *P. delicatissima* Cells

**Figure 5:**
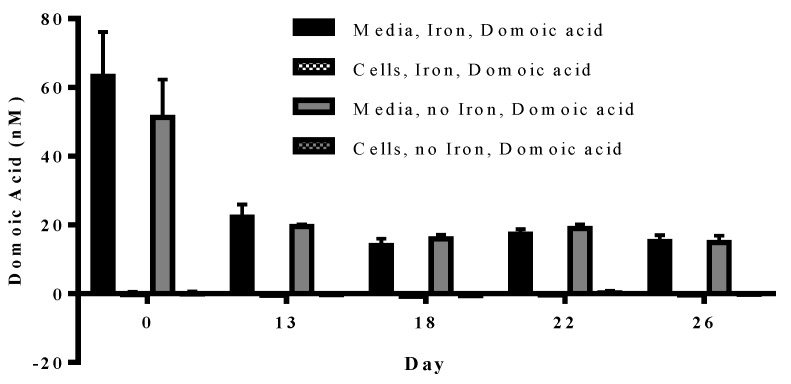
Concentration of particulate (patterned bars) and dissolved (solid bars) domoic acid in *P. delicatissima* cultured under iron replete (black bars) and low iron (grey bars) conditions. Particulate domoic acid was not significantly different from 0. Dissolved domoic acid was not significantly different between iron replete and low iron treatments. However, domoic acid concentration significantly decreased over time (*p* < 0.001). Error bars indicate standard error (*n* = 7 for all).

Experiment 3 tested whether *P. delicatissima* takes up domoic acid under low iron or iron replete conditions in the presence of 63.4 nM domoic acid concentrations. We found that, over the course of the *P. delicatissima* growth curve, the domoic acid content in cells grown in low iron and iron replete cultures was never significantly different from zero (*p* = 0.156–0.938). Although there was no significant difference between the domoic acid concentration within the low iron and iron replete media (*p* = 0.355) or cell (*p* = 0.827) samples, domoic acid concentration did decrease over time (*p* < 0.001) (Figure 5).

Our results suggest that if domoic acid does chelate iron as a mechanism for iron uptake, complexes are dissociated at the cell surface. These results are consistent with the accepted picture of iron uptake by eukaryotic phytoplankton. While bacteria possess specific siderophore receptors to take up siderophore complexed iron [30], eukaryotic phytoplankton are believed to use a plasma membrane ferrireductase [31] that reduces organically bound Fe(III) to inorganic Fe(II). Iron may be taken up either as inorganic Fe(II) or following reoxidation to Fe(III) [13]. If *P. delicatissima* uses a ferrireducatase to access iron bound to domoic acid, domoic acid would remain outside of the cell. Similarly, if domoic acid indirectly helps *Pseudo-nitschia* spp. access iron (e.g., by making copper available for a high-affinity uptake system [18]), domoic acid-copper complexes must also be dissociated at the cell surface, potentially through a cupric-reductase system. Such a system has been reported from the 2–20 μm size class of a natural phytoplankton assemblage [32].

In contrast to the *P. delicatissima* co-cultures, where domoic acid levels were constant, in *P. delicatissima* monocultures domoic acid levels decreased over the course of the experiment. It is not clear what factors were responsible for this difference, although many reasons can be envisaged for the finding. Future studies should investigate the possibility of altered physiological or physicochemical properties of the cells and/or medium in co-cultures. In fact, multiple changes in the endo- and exometabolome have been observed in standard co-culturing experiments, indicating the strong and diverse physiological response of algae to the presence of other species [33].

### 2.4. Concentration of Iron in Experimental Media

The concentration of iron measured in the low iron media was 0.18 μmol L^−1^, nearly two orders of magnitude lower than concentrations in the iron replete media. Other studies [17,18] have used lower concentrations of iron to investigate iron limitation, leaving open the question of whether either of the diatom species was actually iron-limited in this experiment. Additionally, because the concentration of iron in all media, including the low iron medium was roughly two orders magnitude higher than the concentration of dissolved iron in coastal seawater [2] conclusions about whether iron limitation influenced the outcome of experiments should be drawn carefully. However, the low iron treatment is in the range that is limiting for coastal strains of *Pseudo-nitzschia* spp. and other coastal diatoms [9,34]. More importantly, the growth rate of both diatom species was lower in the low iron treatments than in the iron replete treatments. The concentration of *S. marionoi* in co-cultures hovered around starting conditions. Even the growth of *P. delicatissima* was greatly suppressed, reaching only 17%–40% of cells grown in “high iron” media, and remaining in lag phase approximately 10 days longer. Taken together, our results suggest that both species faced iron limitation. Regardless of whether either species faced iron limitation, however, the negative effects of domoic acid on *S. marinoi* co-cultured with *P. delicatissima* are clear.

## 3. Experimental Section

### 3.1. Phytoplankton Culturing

Experiments were performed with two species of cosmopolitan diatoms. *Pseudo-nitzschia delicatissima* (NCMA 1309) was obtained from the Provasoli-Guillard National Center for Marine Algae and Microbiota (NCMA). *Skeletonema marinoi* strain G4 was obtained from Dr. Jens Nejstgaard, collected from Raunefjord, Western Norway. Both species were maintained at 4 °C with a 12 h LD cycle with a Philips Master TL-D 15W-827 bulb, producing an irradiance of 30–60 μmol photons m^−2^ s^−1^ (PAR). Cultures were maintained in L1 + Si media (NCMA) made with filtered seawater from the North Sea off Helgoland, Germany (salinity 32 psu). Polycarbonate bottles used to make media were cleaned with concentrated nitric acid then rinsed with bidistilled water. Media used for experiments was also made with filtered seawater. Iron replete media was L1 + Si media with an iron concentrations of 1.17 × 10^−5^ mol L^−1^ Fe(III) as described by Guillard and Hargreaves [35]. Low iron media was L1 + Si media with no added iron. Because we did not attempt to remove iron impurities from the low iron media, the degree of iron contamination was assessed at 1.79 × 10^−7^ mol L^−1^. Before experiments, all cultures used in low iron treatments were transferred to and grown in low iron media at least three times. Cultures were not axenic and no attempt to quantify the bacteria present was made, however preliminary experiments and data from Figure 4 suggest that the presence of bacteria did not result in degradation of domoic acid.

### 3.2. Experiments

#### 3.2.1. Experiment 1

Experiment 1 tested whether the presence of domoic acid in iron low or iron replete media affected the growth of monocultures of *P. delicatissima* or *S. marinoi*. For each species, we created four treatments: Fe+ (iron replete), Fe+/DA (iron replete with added domoic acid), Fe− (low iron), and Fe−/DA (low iron with added domoic acid). In *P. delicatissima* cultures, 1 mL of a 20,000 cells mL^−1^ stock culture was added to 39 mL of media to a final volume of 40 mL. In *S. marinoi* cultures, 2 mL of a 100,000 cell/mL stock culture was added to 38 mL of media to a final volume of 40 mL. Additionally, 60 μL of a 10 μg mL^−1^ stock solution of domoic acid (Sigma Aldrich) dissolved in water was added to all Fe+/DA and Fe−/DA treatments, for a final concentration of 150 ng mL^−1^ (482 nmol L^−1^) in each culture. The growth of cultures was monitored by transferring 300 μL to a black 96-well plate and measuring fluorescence using a Berthold Mithras LB940 plate reader (excitation, 430 nm; emission 665 nm; lamp energy, 11000, calibrated with a chlorophyll a standard) with MicroWin 2000 software. All measurements are reported as μg L^−1^ chlorophyll a the culture. The total concentration (dissolved and particulate) of domoic acid in each culture was measured on day 0 and day 34 as described below.

#### 3.2.2. Experiment 2

Experiment 2 tested whether domoic acid improves the competitive ability of *P. delicatissima* under iron replete and low iron conditions. We monitored the growth of co-cultures of *P. delicatissima* and *S. marinoi* under low iron and iron replete conditions, with and without added domoic acid. Seven replicate cultures were created for each of four treatments: Fe+ (iron replete), Fe+/DA (iron replete with added domoic acid), Fe− (low iron), and Fe−/DA (low iron with added domoic acid). In each culture, 1 mL of a 38,000 cell mL^−1^ stock culture of *P. delicatissima* and 1 mL of a 165,000 cell mL^−1^ stock culture of *S. marinoi* was added to 38 mL of media, making a final volume of 40 mL in each culture. These cultures were used for a “high” domoic acid treatment, in which 16 μL of a 50 μg mL^−1^ stock solution of domoic acid (Sigma Aldrich) dissolved in water was added to the Fe+/DA and Fe−/DA treatments, making the final concentration of domoic acid in each replicate culture of 20 ng mL^−1^ (63.4 nmol L^−1^). Seven additional replicates from each treatment were created for “Low” domoic acid treatments and controls, which were identical in all ways except that final domoic acid concentration in the Fe+/DA and Fe−/DA was 2 ng/mL (6.34 nmol L^−1^).

Growth of *P. delicatissima* and *S. marinoi* cells in each culture was monitored by chlorophyll a fluorescence as described above. Additionally, on day 0, 7, 14, 18, 24, 30 and 38, 1 mL samples of each culture were preserved in Lugol’s solution. Concentrations of *P. delicatissima* and *S. marinoi* cells were determined by counts using a Fuchs-Rosenthal hemocytometer or a Palmer-Maloney settling chamber with an upright microscope (Leica DM 2000, Leica, Germany). On day 0 and day 38, 7 mL of each culture was frozen for subsequent domoic acid analysis as described below.

#### 3.2.3. Experiment 3

Experiment 3 tested whether *P. delicatissima* was able to take-up extracellular domoic acid. In this experiment, domoic acid was added externally to *P. delicatissima* cultures and measured over the diatom’s growth curve. Under sterile conditions, 5 mL of stock cultures of *P. delicatissima* (12,000 cells mL^−1^) were added to 35 mL of iron replete or low iron media. Domoic acid stock solution was added to each culture as described for “high” domoic acid treatment in experiment 2. Growth of *P. delicatissima* was monitored by chlorophyll a fluorescence (every 1–2 days) as well as by cell counts (day 0, 14, 18, 22, and 26 of the experiment). Particulate and dissolved domoic acid was measured on days 0, 13, 18, 22, and 26. Domoic acid was extracted and analyzed as described below.

### 3.3. Domoic Acid and Iron Extraction and Quantification

Domoic acid was extracted from whole cultures and media using a modified version of the procedure described by de La Iglesia [36]. To analyze whole culture samples, 5 to 10 mL of culture was frozen to lyse the diatom cells and stored until analysis. To analyze particulate and dissolved domoic acid separately, we filtered 5 mL of culture through a 24 mm GF-C filter (Whatmann). The media was transferred to a 15 mL falcon tube. Both media samples and filters were stored at −20 °C until analysis. In order to concentrate dissolved domoic acid, the pH of each sample was adjusted so that it fell within a range of 1.85 to 4.47 (*i.e.*, the range at which domoic acid has a net neutral charge). Next, each sample was loaded onto an Oasis HLB 1cc cartridge (Waters). Domoic acid and other organic metabolites were eluted from the column with 750 μL of 9:1 water:acetonitrile (pH adjusted with NaOH to >8) followed by 750 μL of methanol. The eluant was collected in a 1.5 mL vial. Preliminary tests indicated that 75%–95% of dissolved domoic acid was extracted through this method (data not shown). Particulate domoic acid was extracted from cell filter samples. In order to ensure cell lysis, each filter was vortexed in 750 μL of methanol for 1 min. Next we added 750 μL of bidistilled water, and vortexed for an additional minute. Samples were centrifuged for 15 min at 6000 rpm at room temperature, and the supernatant was transferred to a 1.5 mL vial. All samples were analyzed as described below. If domoic acid concentration was below the concentration of lowest standard concentration used for the generation of the standard curve, samples were concentrated under vacuum to 150 μL and reanalyzed. Quantification of domoic acid was performed using an Acquity ultra-performance liquid chromatography (UPLC) coupled to a Waters time of flight Q-ToF micro-mass spectrometer with electrospray ionization (ESI). For each sample 20 μL was injected onto a C18 UPLC column (5 cm length, 2.1 mm diameter, 1.7 μm particle size, Waters) at 30 °C. Domoic acid was separated using an acetonitrile/water gradient (0.1% formic acid). The solvent gradient began with 98% water, 2% acetonitrile and was constant for 0.2 min. The concentration of acetonitrile increased gradually to 100% at 3.0 min, remained constant until minute 3.5, decreased to starting conditions by minute 4.0, and was held at those conditions until minute 5.0. Mass spectra were recorded in negative mode, with a scan range from 300 to 350 *m/z*. Domoic acid concentration was determined by comparison to a linear standard curve (obtained with six domoic acid standard solutions with concentrations between 10 ng mL^−1^ and 1 μg mL^−1^;. Responses for domoic acid specific ions ([M − H]^−^ *m/z* 310 and [M − 2H + Na]^−^ *m/z* 332) were integrated using Waters MassLynx software, and concentrations were determined by interpolation from a linear regression using Microsoft Excel. Iron was determined using inductively coupled plasma optical emission spectrometry (ICP-OES) by IAU (Neuhaus, Germany).

### 3.4. Statistical Analysis

In order to determine the effects of treatment on cell growth over time, we used a two-way repeated measures ANOVA (SigmaPlot 11.0) for experiments 1 and 2. Effects were accepted as significant when *p* ≤ 0.05. In order to determine which treatments were significantly different from each other and on which days, the Holm-Sidak method was applied. Results were accepted as significant when the *p*-value of comparisons was lower than the *p*-value of the Sidak adjustment (SigmaPlot 11.0). For experiments 1 and 2, changes in domoic acid over time and differences in domoic acid concentrations between treatments were also analyzed by a two-way repeated measures ANOVA with a Holm-Sidak post-test (SigmaPlot 11). For experiment 3, we determined if concentrations of domoic acid were significantly different from zero using a one-sample *t*-test (GraphPad Prism 5). Results were accepted as significantly different from zero when *p* ≤ 0.05. Effects of time, treatment, and the interactions between time and treatment, were determined using a two-way repeated measures ANOVA.

## 4. Conclusions

We found that domoic acid can improve the competitive ability of *P. delicatissima* over the cosmopolitan diatom *S. marinoi*. Our results cannot be explained by a direct toxic effect of domoic acid on *S. marinoi* because, although high concentrations of domoic acid can slightly inhibit *S. marinoi* growth under low iron conditions, even very high concentrations of domoic acid had no effect on the growth of *S. marinoi* under iron replete conditions.

Overall, our results provide insight in to competition between marine diatoms, and provide an example indirect inhibition of competitor growth via a secondary metabolite. Although the mechanism for this effect remains unknown, our results, coupled with previous studies, suggest that *Pseudo-nitzschia* spp. may use domoic acid as part of an iron uptake system that might render iron unavailable for the competitor. By inhibiting the growth of other phytoplankton, albeit indirectly, through the production of domoic acid, *Pseudo-nitzschia* spp. is likely to alter the composition of the phytoplankton community. Domoic acid, which can directly alter ecosystem functioning through its toxicity, may also have more subtle, indirect effects on higher trophic levels by changing the availability of prey.
